# Obstructive Sleep Apnea and Its Management: A Narrative Review

**DOI:** 10.7759/cureus.37359

**Published:** 2023-04-10

**Authors:** Vrushabh G Gomase, Prasad Deshmukh, Vedant Y Lekurwale

**Affiliations:** 1 Otorhinolaryngology, Jawaharlal Nehru Medical College, Datta Meghe Institute of Higher Education and Research, Wardha, IND; 2 Otolaryngology and Head and Neck Surgery, Jawaharlal Nehru Medical College, Datta Meghe Institute of Higher Education and Research, Wardha, IND; 3 Medical School, Jawaharlal Nehru Medical College, Datta Meghe Institute of Higher Education and Research, Wardha, IND

**Keywords:** obstructive sleep apnea (osa), oxygen desaturation index, positive airway pressure, snoring, polysomnogram

## Abstract

Obstructive sleep apnea (OSA) is a disorder in which there is repeated collapse of the upper airway when the person is in sleep, which causes oxygen desaturation and interrupted sleep. While asleep, airway blockages and collapse are accompanied by awakenings with or without oxygen desaturation. OSA is a prevalent disorder, especially in people with known risk factors and other illnesses. Pathogenesis is variable, and the risk factors include low chest volume, erratic respiratory regulation, and muscular dysfunction in the upper airway dilators. The high-risk factors include overweight, male sex, aging, adenotonsillar hypertrophy, interruption of the menstrual cycle, preservation of liquids, and smoking. The signs are snoring, drowsiness, and apneas. A sleep history, assessment of symptoms, and physical examination are all part of the screening process for OSA, and the data can help determine which people need to be tested for the condition. The results of the polysomnogram or at-home sleep apnea test assist in determining the presence and severity of OSA. Still, it is seen many times that the accuracy of home sleep apnea tests is significantly less, so one should take an expert opinion for the same. OSA results in systemic hypertension, drowsiness, and driving accidents. It is additionally related to diabetes mellitus, congestive heart failure (CHF), cerebral infarction, and myocardial infarction, but the exact mechanism is not known. The preferred treatment is continuous positive airway pressure with 60-70% adherence. Other management options include reducing weight, therapy of oral appliances, and correcting any anatomical obstruction (narrow pharyngeal airway, adenoid hypertrophy, and mass in the pharynx). OSA indirectly causes headaches just after awakening and daytime sleepiness. However, there are no age boundaries in OSA as it can occur in any age group. Still, more prevalence is seen in individuals of more than 60 years of age.

## Introduction and background

Obstructive sleep apnea (OSA) is characterized by repeated episodes of upper airway blockage and collapse during sleep, accompanied by awakening with or without oxygen desaturation. Common symptoms include excessive daytime sleepiness, fatigue, non-refreshing sleep (sleep that is insufficiently refreshing), nocturia, morning headache, irritability, and memory loss [1]. When OSA events occur, the oropharynx in the posterior aspect of the throat collapses, causing arousal, oxygen desaturation, or both, leading to disturbances in sleep. OSA occurs when the muscle that supports the soft muscles in the deeper part of the mouth, such as taste buds and soft palate, temporarily relaxes. When these muscles relax, the airway is almost closed completely and breathing is nearly cut off. Obesity is also considered as one of the main reasons for OSA. Obesity causes narrowing of the respiratory airway leading to hypoxic and apneic episodes resulting in sleep apnea.

Objective

The primary goal of this article is to determine the alarming symptoms, treatment options, and preventive measures of OSA. 

## Review

Methodology

**Figure 1:**
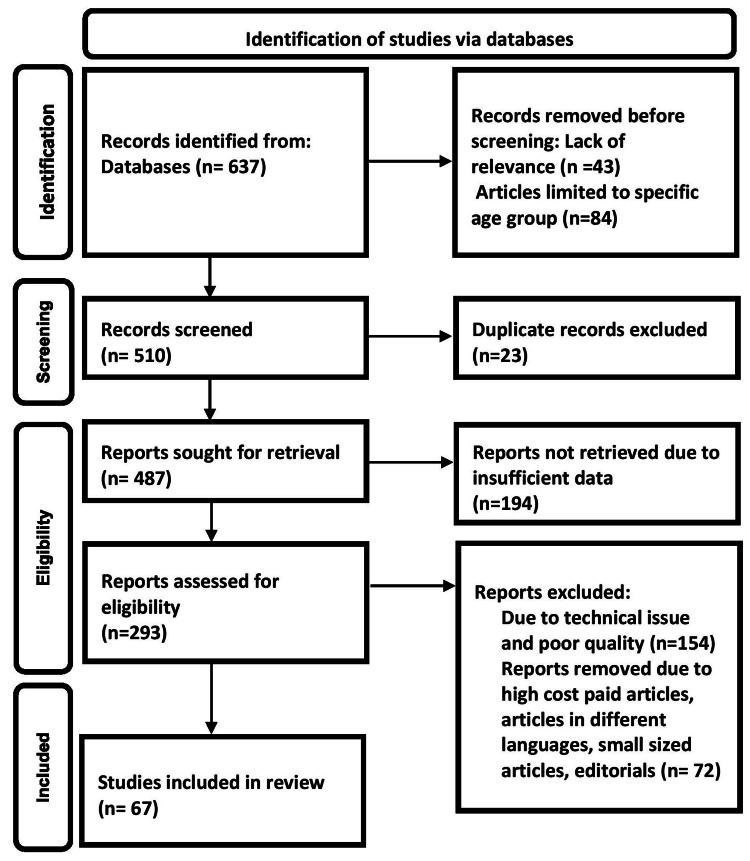
PRISMA model for search strategy PRISMA, preferred reporting items for systematic reviews and meta-analyses

OSA, oxygen desaturation, polysomnogram, snoring, and positive airway pressure were the search terms utilized in Embase, 80 articles; Scopus, 118 articles; Cochrane, 94 articles; Google Scholar, 107 articles; and advanced PubMed database, 238 articles. The search turned up 637 papers, and 66 research publications were chosen for further research. Figure 1 illustrates the PRISMA method's technique. PRISMA stands for preferred reporting items for systematic reviews and meta-analyses.

There are two types of pharyngeal fall-outs, total and near-total hypopnea. This disturbance causes hypercapnia, oxygen desaturation, and other conditions in gaseous exchange. Disturbances in sleep are linked to OSA's adverse effects, such as its impact on the heart, metabolism, and brain function. Although there are numerous therapies, these therapies partially relieve problems or are poorly tolerated; therefore, enhancing patient compliance with current treatments and creating new ones are preferred. Sometimes, the application of more than one therapy (or therapy combinations) is required. Obesity increases the tendency of pharyngeal collapse, which can increase the prevalence of OSA. A seminal investigation was done on OSA in the year 1993. According to the Wisconsin Sleep Cohort Study [2], 4% of middle-aged men and 2% of middle-aged women (ages 30-60 years) had OSA, which is defined as having more than five episodes of apneas or hypopneas in each hour of sleep with severe drowsiness even during the afternoon.

Risk factors 

Both non-modifiable and modifiable factors that affect the risk of OSA are shown in Table 1. Risk factors that cannot be modified include race, age, and male sex. A higher risk of OSA may be associated with cranio-facial structures that result in restricted airways, genetic susceptibility, and a family history of OSA. Modifiable risk factors include obesity, drugs that relax muscles and constrict the airway (opioids, benzodiazepines, and alcohol), endocrine conditions (hypothyroidism and polycystic ovary syndrome), smoking, alcoholism, and nasal congestion or obstruction [3].

**Table 1:** Risk factors for obstructive sleep apnea

Non-modifiable risk factors	Modifiable risk factors
Race	Obesity
Age	Muscle relaxant drugs
Sex	Endocrine conditions
Cranio-facial defects	Smoking
Genetic susceptibility	Nasal congestion
Family history of obstructive sleep apnea	Alcoholism

Comorbidities

Stroke, myocardial infarction, and other concurrent conditions are all linked to OSA. Hyperlipidemia, hypertension, glucose intolerance, diabetes, atrial fibrillation, pulmonary hypertension, CHF, depression, and arrhythmia are other comorbidities that are linked to OSA. Patients with cardiovascular disease have a very high prevalence of OSA, such as hypertension (83% mild to 30% moderate to severe OSA), heart failure (55-12%), arrhythmias (50-20%), stroke (75-57%), and coronary heart disease (65-38%) [4].

Diagnosis

Snoring, witnessing apneas, waking up with a headache, extreme tiredness, and choking sensation are all reported by patients with OSA. Lethargy or exhaustion, inability to get sleep or stay asleep, and morning headaches are also seen. A specific characteristic suggestive of OSA is the tiny airway between the mouth and the pharynx. Other indicators of OSA include being overweight (such as big neck circumference) [5].

Overnight polysomnography in a lab with an air pressure monitor is the test recommended for OSA. The apnea-hypopnea index (AHI) is the key performance indicator (no. of apneas + hypopneas/hour of sleep). Both sleep and breathing are being monitored simultaneously throughout this test. Electroencephalogram, left and right electrooculogram, and chin electromyogram readings are taken to track the sleep-wake state. The respiratory recordings should include arterial oxygen saturation, nasal air pressure, thermal air sensors to monitor airflow, and respiratory inductance plethysmography bands to evaluate breathing effort. Due to the position-specific nature of OSA in many individuals, electric myography of the anterior tibialis muscle has been frequently performed to check limb motions that can affect sleep or respiration.

Although polysomnography has been typically conclusive, the process is laborious, expensive, and time-consuming. As a result, research into home diagnosis and treatment has increased the home-based identification of conditions and management compared to lab diagnosis and management for a few patients, according to randomized controlled trials [6-9]. To emphasize that management at home isn’t appropriate for all patients, which includes potentially complex patients (such as those with bronchial illness, myocardial infarction, or neuromuscular disease). Ongoing studies concentrating on clinical results assist in defining the ideal management of OSA. Still, for a few patients, well-planned home remedial protocols like reduction in weight, yoga, and altering the sleeping position can give timely management of the condition at a low cost [6].

Pathophysiology 

Traditionally, upper respiratory issues were thought to be the leading cause of OSA. The airway, which is affected by obesity or cranium-facial shape, reduces the size of the lumen of the pharyngeal airway, increasing the risk of pharyngeal collapse [10]. The intense activity of the various upper respiratory muscles keeps the airway open while a person is awake. However, when sleep takes hold and muscular activity declines, the upper respiratory muscles fail to keep the airway open; this sequence most likely happens in OSA, and several more elements also play a role [11]. The significance of those nonanatomical, neuromuscular factors is demonstrated in individuals having supposedly healthy upper airways, in which highly responsive dilatory muscles of the upper airway are also developing obstructive snoring [12]. The respiratory control system's stability is one crucial factor. OSA is most likely caused by obstruction of the airway; due to this obstruction, the activity of the muscles that dilate the upper airway is altered and is linked to increased airway resistance, airway dilator muscle movement, and a propensity for collapsing airways [12-15].

The propensity to awaken from sleep is another element that could be significant (the awakening threshold) [16]. Following arousal, most people quickly hyperventilate, and if blood concentrations of carbon dioxide do not drop below the threshold of chemical apnea, it will cause central apnea [17]. If hypocarbia is moderate, the respiratory level is decreased to just below the vital threshold so that sleep is resumed. Since respiratory muscles that dilate the upper airway also receive respiratory input, hypocapnia will lead to less action of the muscles that dilate the upper airway and may obstruct the airway. Respiratory episodes in people with OSA are stopped by arousals that are frequently accompanied by extremely severe hyperventilation due to increased respiratory drive significantly throughout the respiratory episode, resulting in hypocapnia in at least some individuals.

In these circumstances, non-muscle-relaxing sedatives may be used to delay arousal. These sedatives can be used if the upper airway muscles are receptive enough to the treatment; it may be respiratory stimulation to secure the airway before awakening [18]. We must take caution with this strategy, as sedatives may cause certain patients’ respiratory episodes to last longer.

Another potential contributing component to OSA is lung volume [19,20]. In humans and animals, the cross-sectional lung capacity increases when the upper airway region expands. In contrast, if the airway is narrower and a person has a small lung volume, the airway will collapse more quickly [21]. This relationship likely exists due to the mechanical connection between the lower and upper airways [22]. When lung volume increases, the caudal pulling of mediastinal tissues causes stiffening and dilatation of the airway of the pharynx. The respiratory control is likely stabilized by increased lung volume and by boosting the oxygen and carbon dioxide reserves in the system [23]. Any elements, such as malfunctioning muscles that affect the architecture or functionality of the muscles of the upper airway that dilate the airways, will also intensify the risk of OSA [24]. The genioglossus is the biggest & well-researched inspiratory muscle, making up most of the tongue. Adequate contraction of the genioglossus muscle is required to maintain the upper airway wide while sleeping [25]. Unhealthy people with obstructive pulmonary disease may experience genioglossus dysfunction due to its damage and myopathy, resulting in snoring [26-28]. Pharyngeal sensory dysfunction could potentially result in the collapse of a higher airway [29,30]. Major risk factors for OSA include being male and being obese. Obesity may directly alter the anatomy of the upper airway to raise the risk of airway collapse as fat is accumulated in neighboring structures [31] within the tongue, making the genioglossus muscle less effective [32]. Through its impact on lung volumes, obesity may raise the risk of OSA and thus alters the proper maintenance of breathing. The link between OSA and the male sex is not clear yet [33]. Men often acquire weight more centrally than women, and these men are likely to have more fat accumulated around the upper airway structures than women [34].

Another significant risk factor is age. Older people may have less lung volume as the lung parenchymal tissue is decreased with age. They could also have an airway that is more susceptible to collapsing due to collagen loss. Lastly, the dilatory function of upper airway muscles weakens as we age [35-37]. Obesity (body mass index of over 30 kg/m2) correlates to OSA, including a larger waist-to-hip ratio and neck circumferences, which are also associated with an increased risk of OSA [38]. A 10% increase in body weight causes a sixfold increase in moderate to severe OSA and increases the AHI by 32%, while a 10% decrease in weight reduces the AHI by 26% [39].

Management

For individuals, continuous nasal positive airway pressure is the preferred treatment method. Constant positive airway pressure was discovered in 1981 and was initially identified as a valuable method of preventing a collapse in 1998 [40]. But most likely, keeping a positive pharyngeal pressure across the vessel wall so that the intraluminal pressure is greater than the ambient pressure is beneficial [19]. End-expiratory lung capacity increases due to airway pressure, which helps in stabilizing the upper respiratory tract. Before starting the therapy with continuous positive airway pressure, talking with the patient regarding the potential relief from airway pressure signs and potential cardioprotection is necessary [41-43].

Several factors should be considered when managing patients who cannot follow instructions while performing persistently positive airway pressure. First, intensive assistance can be helpful [44]. Second, a few patients may experience nasal issues restricting the tolerance for continuous positive airway pressure nasal therapy. For certain patients, decongestants and warm humidification may be helpful. On rare occasions, surgery on the nose can increase compliance [45]. Third, despite the lack of evidence from randomized trials, some patients recommend a full face mask over a nasal mask. People can also choose a nasal cushion device. Fourth, hypnotherapy may be effective for some patients if they either get insomnia or wake up a lot when they use continuous positive airway pressure. Eszopiclone can be used initially for those who experience sustained positive force in the airways [46]. Once the patient has become accustomed to the new technology, hypnotherapy is no longer required. In patients with OSA, sedatives should be used with caution.

Patients with persistent positive airway pressure have several different choices. Bilevel positive airway pressure (Bi-PAP) is a possibility (and can be employed) and is favored by a few patients experiencing pain from expiratory pressure, even while randomized trials have not revealed any significant advantages over continuous positive airway pressure [47]. Second, techniques for relieving expiratory stress, such as C-Flex, can lessen discomfort in some patients. The majority of data indicate that such treatments don't provide many benefits [48] compared to conventional continuous positive airway pressure. Oral devices, surgery of the upper airway, positional therapy, and other prophylactic measures are options for people in whom positive airway pressure fails. Although dental devices also function in a variety of ways, they primarily provide pressure to the jaw to preserve their retroglossal stability. Continuous positive airway pressure is not as effective as oral devices. Some individuals, especially those with mild to moderate illness, may experience anxiety [49,50]. The effectiveness of oral machines differs from person to person [51-54].

Rarely soft palate can undergo straightforward operations (somnoplasty and a laser-assisted uvulopalatopharyngoplasty) [55]. However, the relief from symptoms is typically just temporary in most cases. However, less than 50% of patients see significant improvement in AHI. Hence, many medical professionals advise against doing the uvulopalatopharyngoplasty procedure. However, because surgical methods eliminate patient-related challenges, some researchers recommend surgical procedures for adherence [55-58]. Conservative actions are also sometimes beneficial. Avoiding CNS depressants like alcohol and smoking can be helpful, as these may worsen symptoms. Seven to eight hours of sleep every night can lessen drowsiness. Patients with OSA can benefit from avoiding a supine posture. These patients frequently use a continuous favorable airway positioning treatment under pressure [59]. Positional treatment can also be challenging, and clinical outcomes are unpredictable because of home monitoring. Positional therapy is also done with other medicines and combined with interventions like oral devices or surgery, producing ambiguous results. They can also be helped by losing weight by exercising and having a proper diet [60].

Prevention

Although many risk factors for OSA are fixed, reduction of weight, avoiding tobacco, alcohol, and other muscle-relaxing medications, as well as regular exercise, can be helpful [60,61]. A randomized controlled trial's findings indicate that a 10 kg decrease in body weight can result in a five to 10 events per hour drop in the apnea-hypopnea score [62]. Compared to those with a severe condition, only 13% of those with mild disease had OSA. People with severe OSA get remission by losing weight, and reduction in episodes of apnea is highly effective if weight loss is considered [62]. OSA recurs even after surgical or nonsurgical weight reduction [63-67].

## Conclusions

OSA is common in people of age more than 60 years with male preponderance. However, it can be present in any age group. It is linked to various risk factors, of which obesity plays a major role. Incidences of OSA will rise globally in the upcoming time due to obesity and the aging population. Continuous positive airway pressure and weight reduction have shown promising effects in reducing the episodes of OSA. Weight loss, alcohol restriction, and muscle-relaxing drugs have shown positive results in controlling OSA. More progress is seen in people who have reduced their weight by 10 kg.
